# Robotic applications: lessons on what constitutes success

**DOI:** 10.1155/S1463924691000032

**Published:** 1991

**Authors:** Beth Hutchins

**Affiliations:** Genentech, Inc. Department of Medicinal and Analytical Chemistry Biological Chemistry Section South San Francisco California 94080 USA


Journal of Automatic Chemistry, Vol. 13, No. 1 (January–February 1991), pp. 9–12

### Robotic applications: lessons on what constitutes success*

**Beth Hutchins**
*Genentech, Inc., Department of Medicinal and Analytical Chemistry, Biological Chemistry Section, South San Francisco, California 94080, USA*

### Introduction

As for all things, laboratory robotic applications are judged as to whether they are successful. Some criteria used to measure success are the ability to design and program the instrumentation, reliability and accuracy during operation, and cost reduction resulting from using the robotic method rather than a manual method. Although all of these factors are important contributors to the success of a robotic application, the author's experience is that the best indicator of the success of a robotic application is its acceptance and frequent use by the intended user(s).

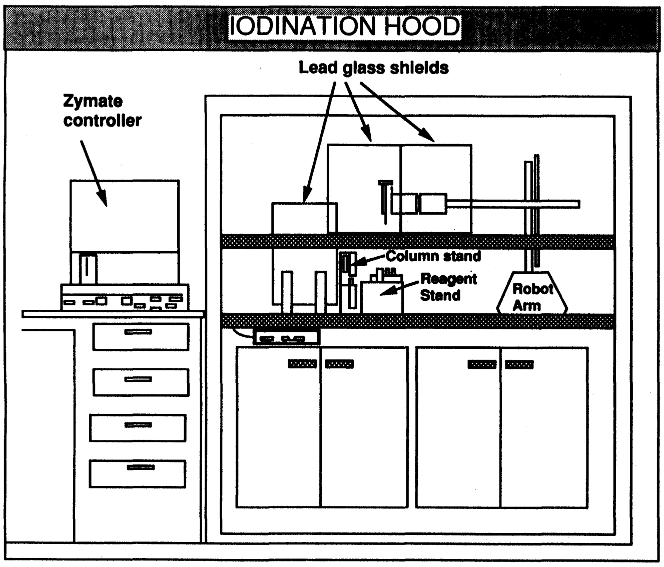

(*a*)

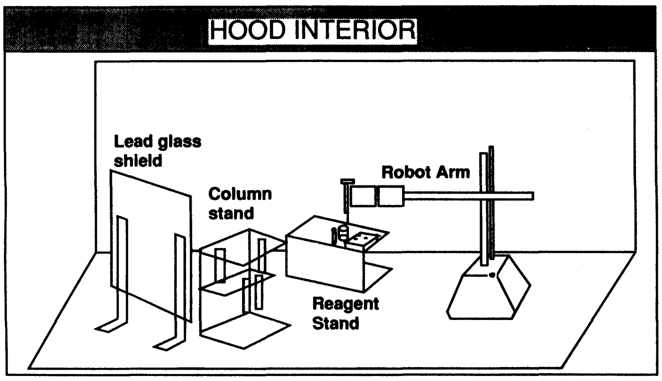

(*b*)

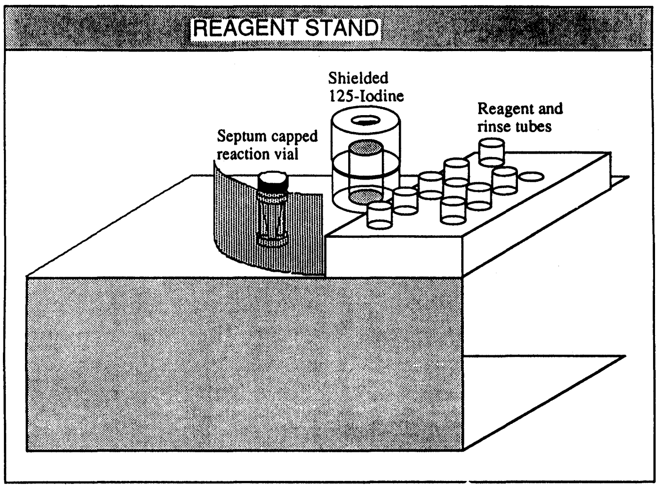

(*c*)

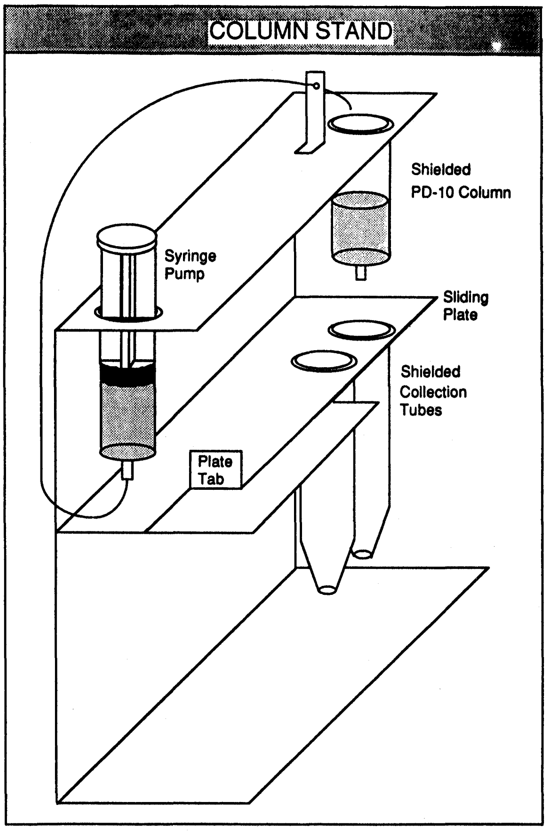

(*d*)

*Figure 1. Robotic system for iodinations. (a) Front view: except for the controller, the system sits within a fume hood. (b) Hood interior overview: indicates positions of robot arm, reagent/reaction station and column stand. (c) Reagent stand detail: both the reaction and source iodine vials are septum-sealed and shielded. The reaction reagents and rinse tubes are also located here. (d) Column stand detail: after application of the reaction mixture to the column, the robot pushes down on the syringe to deliver a measured quantity of buffer onto the column. The labelled protein is collected from the column in tubes. The robot slides the plate holding the collection tubes into position by pushing the 'plate tab'.*

* Abstract published in *Journal of Automatic Chemistry*, Vol. 12, No. 6.

*Table 1. Comparison of an RIA tracer prepared manually and robotically.*

| Method[a] | Specific activity (μCi/μg)[b] | Percentage immuno-precipitability[b] | Kd determined for standard[b] |
|---|---|---|---|
| Manual | 30·6 | 95·3 | $1{\cdot}04 \times 10^{-10}$ |
| Robotic | 60·1 | 98·9 | $0{\cdot}95 \times 10^{-10}$ |

[a] Iodination of the tracer receptor protein was performed using the chloramine-T method. Percentage immunoprecipitability was determined using a polyclonal antisera.
[b] Although there was a difference in the specific activity of the two preparations, there were no observed differences in the performance of the two lots, as demonstrated by the Kd of the standard.

At Genentech varying degrees of success have been encountered in laboratory applications. The purpose of this paper is to identify the elements of those applications considered successful and to understand the problems in an example of a less than successful application.

### Automated iodination of proteins

One 'success' story is the application of robotics to automating the iodination of proteins and peptides by a variety of chemistries. In this system the robotic components are merely used as a substitute for the person's hand in the manual method. Figure 1 displays schematic representations of Genentech's robotic system for iodinations. This system, with the exception of the controller, sits within a fume hood outfitted for radioiodinations. A dual-purpose syringe/gripper hand is used to transfer the coupling reagents and radioactive iodine to septum-sealed reaction vial containing the protein. The syringe is then used to transfer the reaction mixture to a small column in order to separate the radiolabelled protein from the free iodine. The robot completes the chromatography step by delivering buffer onto the column by having the hand push down on a syringe thus causing buffer to flow through the tubing onto the column. The robot then collects fractions from the column by pushing a sliding holder which places the appropriate tube under the column outlet.

*Table 2. Delivery performance of robot syringe.**

| | Nominal μl | % nominal delivered | % CV |
|---|---|---|---|
| Deliver | 10 | 101·8 | 1·49 |
| | 20 | 102·6 | 1·01 |
| | 50 | 102·1 | 0·26 |
| Deliver and mix† | 10 | 108·4 | 2·46 |

* Hamilton 50 μl type RN syringe.

† Mixing = drawing 20 μl in and out of the syringe three times after delivery.

Users begin the procedure by selecting the reaction parameters presented on the controller screen. These include: selection of one of three iodination methods they will be using; the amount of iodine to be used; and the reaction time. The program prompts the user on the appropriate set-up, and will only start the reaction after the user inputs the go-ahead. The selected method (chloramine T, lactoperoxidase, or iodogen) is begun by adding iodine and other labelling components to the reaction vial with mixing. The reaction is incubated for the appropriate time, then, depending on the protocol, stopping or other reagents are added as required. The reaction mixture is then transferred to the column in order to separate the labelled tracer from the free iodine. Finally, the robot washes out the hand syringe so that the user can easily clean up by disposing the column and tubes.

The user is still required to remove radioactive waste from the system and to monitor it after use for any contamination. Contamination, however, is minimized by the use of the septum-sealed reaction vial and source iodine vials. Contamination of the robot surfaces from radioactive aerosols is also minimized because the robot arm surfaces are protected by easily-replaced plastic. Except for the robot hand, all other exposed surfaces in the hood are made of stainless steel and readily decontaminated. The robot hand syringe and needle are routinely replaced to reduce exposure to those running and maintaining the system.

This system is a successful application because it is in constant use. Why is it well utilized? Why is it deemed successful?

First, the system performs in an acceptable manner. It produces radiolabelled proteins that are equivalent to those produced manually. Many different proteins have been iodinated repeatedly using this system in its first 4 months of operation. All have met the quality requirements set for each under the manual methods. Also, the system was set up to handle the most common methods and scales employed. Table 1 shows comparative data from a robotic and a manual iodination of a recombinant receptor protein. In addition, the iodinations performed using the robot have been very reproducible for any given protein and method. This appears to be due to the highly reproducible reagent delivery from the robot hand syringe (table 2).

Second, the system is easy to use. The user places tubes and solutions in the designated areas, uses a menu to select reaction parameters, and sets the system to 'run'. At the end of the procedure, the user removes a few items from the hood and performs the check-out survey.

A third point is that the robotic system is a 'hands-off procedure', which means that a user's exposure to radiation is reduced. Users do not handle the iodine source vial and they do not handle the reaction mixture until after the free iodine has been removed.

Finally, one more measure of the iodination robot's success is that during an 'open-house' period, where employees from other departments were invited to perform iodinations using this system, considerable interest was created in developing such systems for other departments, even with the nearly $35 000 price tag and one person–month labour investment required for set-up.

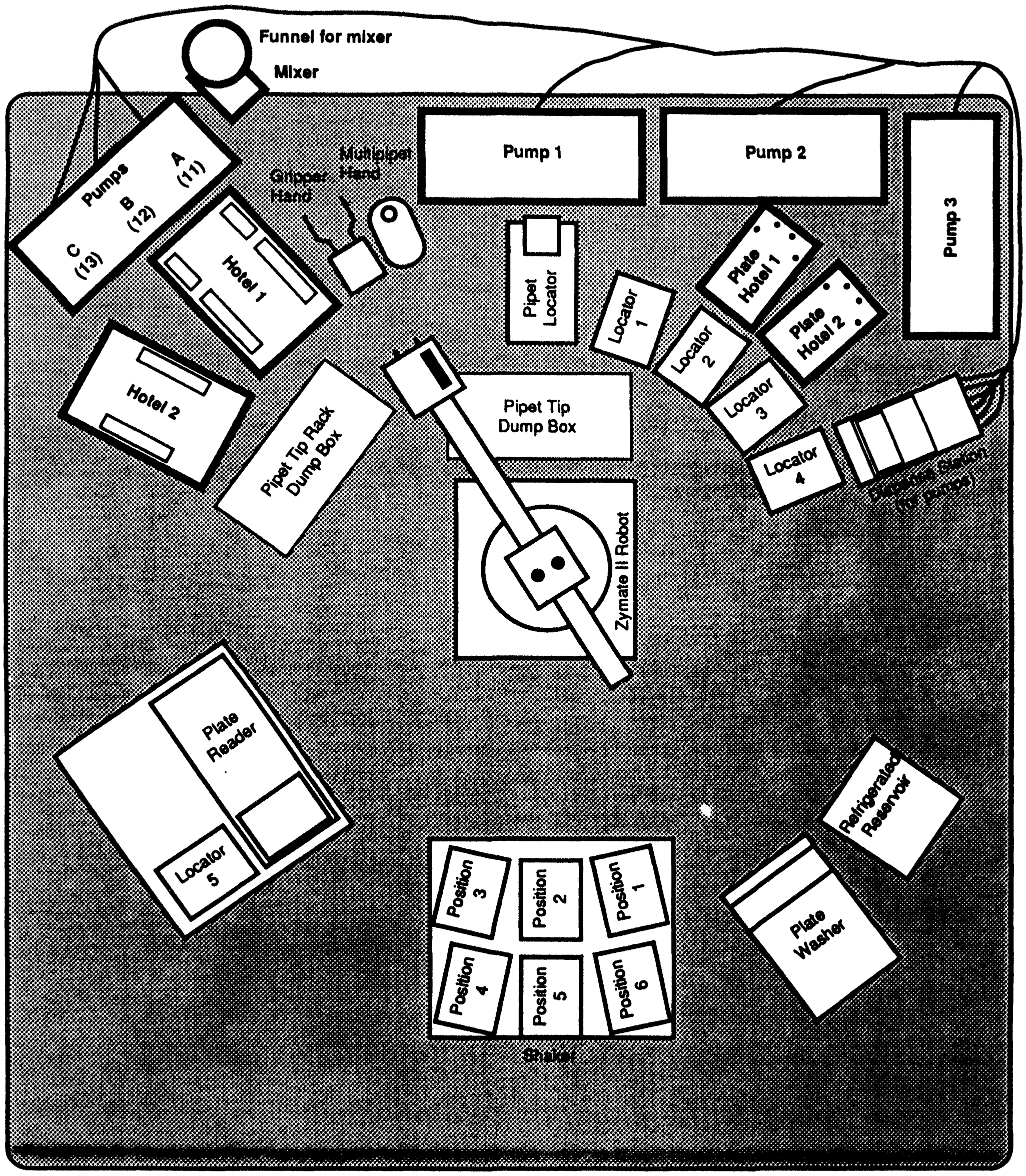

*Figure 2. Microplate management system. Overview of the microplate management system in the methods development laboratory. This system consists of a Zymate II Robot and controller equipped with many custom modules, including a modified Skatron plate-washer (Skatron, Inc., Sterling, Virginia), a modified SLT EAR 340 AT plate-reader (SLT LabInstruments, Hillsborough, North Carolina), and several Zymark liquid reagent pump modules. The system uses both gripper as well as an eight-channel multipipettor hands (Zymark). The controller and master PC sit on a separate table. The system was designed to handle Flow micronics tubes as well as microtitre plates.*

### Robotics in research and development laboratories

Experiences at Genentech with two other robotic applications are also worth noting. Both are Zymark microplate management systems and were each designed to perform several assays, since in research and development laboratories most projects require a degree of experimental flexibility. These systems have very similar, although not identical designs. The peripherals include plate washers, readers and liquid reagent addition stations.

One of the systems (schematic overview in figure 2) resides in a methods development laboratory. In this setting methods are established, and subsequently usually transferred to other groups within the company. Since many projects are transient and have specific objectives, the proteins being studied and details of assay protocols change frequently. This microplate management system is able to perform a variety of procedures. In this system the Zymate controller is a slave to a master program run on a PC. The master program, which was written in-house, was designed modularly so that it could be easily modified to accommodate the constantly changing assay protocols. In addition, it is an interactive menu-driven system, and thus easier for personnel to use the system with minimal assistance.

This system has been shown to perform comparably to manually run assays, and, because of its capabilities, it could be considered successful. This system is, however, only sporadically used, generally for short-term, high-throughput projects. Most personnel in the laboratory have remarked that they feel it is not worthwhile using the robot for assays with only a small number of microtitre plates (less than five).

The other microplate management system resides in a research laboratory. This system is used to screen large numbers of samples weekly, even though any, or all, of five different assays may be required. A single person 'uses' this system, running it as a service to others. The fact that this is a 'dedicated' system, one that is used for one purpose, is the major reason that it is successful, even though it was designed for flexibility.

### Common threads to success

What are the features that the 'successful', well utilized systems have in common? In the case of each of the successful robotic systems, *a specific need was identified* and end-users participated closely in the development process, so the application was carefully defined. In the case of the microplate management system in the methods development laboratory, the purpose of the system was never completely identified. While it has served very well as a methods development tool for other robotic systems, a routine application in an immunoassay development environment has still to be identified.

Another essential feature for success is careful determination of whether it makes sense to use robotic means to accomplish the task. In the case of iodinations, the added measure of safety in reducing laboratory personnel exposure to gamma radiation was worth the set-up time and capital investment. In the case of the methods development laboratory, the personnel felt it did not 'make sense' to use the robot to perform assays with relatively few samples. Where large numbers of samples are involved, laboratory personnel seek relief from the tedium and readily use the robotic system.

Finally, there is the issue of who is using each system. In the screening microplate management system, there is only one person using the robotic system. In this case, 'users' submit samples for assay. In the methods development laboratory system there are many potential users, although only one person has the responsibility to maintain the system. In this case, it appears that the barrier to learning to use the system within the daily routine of the 'users' and changing established habits is too high.

In the case of the iodinations robot, many different individuals use the system, but the system is maintained and all users are trained by one person. Here automation is accepted and therefore used because it relieves the user of the task of concentrating on each specific step in an iodination.

The lesson here is that beyond the ability to get the system to perform its assigned task, what contributes most to the success of a system is the careful definition of the objective of the project prior to its implementation.

